# Associations Between Specific IADLs And Objective Cognitive Performance In Older Women With HIV

**DOI:** 10.1093/geroni/igaf122.2673

**Published:** 2025-12-31

**Authors:** David Vance, Lang Lang, Raha Dastgheyb, Yanxun Xu, Leah Rubin

**Affiliations:** University of Alabama at Birmingham, Birmingham, Alabama, United States; Johns Hopkins University, Baltimore, Maryland, United States; Johns Hopkins School of Medicine, Baltimore, Maryland, United States; Johns Hopkins University, Baltimore, Maryland, United States; Johns Hopkins University School of Medicine, Baltimore, Maryland, United States

## Abstract

Nearly 30% of people with HIV experience suboptimal cognitive functioning, which is expected to increase as people with HIV age. A subgroup particularly at risk for cognitive impairment is women with HIV which comprise 21% of the HIV population in the United States and 53% of the HIV population worldwide. These cognitive impairments can develop into everyday functional impairments. In the Women’s Interagency HIV Study (WIHS), we examined the association between the self-rated Lawton and Brody scale of Independent Activities of Daily Living (IADL) and objective cognitive test performance in 754 older (50+) women with HIV (WWH; 84% virally suppressed). In the total sample, 73% were non-Hispanic Black (mean age = 55.03 years). To handle this longitudinal data, weighted logistic mixed effect models examined associations between cognitive domain performance (predictor) and functional outcomes (IADL item level scores). With respect to IADLs, approximately 38% of the total sample showed an overall impairment in IADLs; however, as expected this impairment (35%) was slightly less in those virally suppressed. In the total sample, poorer executive functioning was associated with impairment in planning social activities and poorer motor performance was associated with impairments in home repairs, housekeeping, and laundry. Among older virally suppressed-WWH, poorer motor performance was associated with deficits in home repair and poorer executive performance was associated with deficits in planning social activities. Since motor and executive performance were related to impairments in certain IADLs, strategies such as cognitive training targeting these domains could improve everyday functioning.

